# Association of Common Genetic Variants in the *MAP4K4* Locus with Prediabetic Traits in Humans

**DOI:** 10.1371/journal.pone.0047647

**Published:** 2012-10-18

**Authors:** Tina Sartorius, Harald Staiger, Caroline Ketterer, Martin Heni, Fausto Machicao, Adilson Guilherme, Harald Grallert, Matthias B. Schulze, Heiner Boeing, Norbert Stefan, Andreas Fritsche, Michael P. Czech, Hans-Ulrich Häring

**Affiliations:** 1 Institute for Diabetes Research and Metabolic Diseases of the Helmholtz Centre Munich at the University of Tübingen, Tübingen, Germany; 2 German Center for Diabetes Research (DZD), Neuherberg, Germany; 3 Division of Endocrinology, Diabetology, Angiology, Nephrology and Clinical Chemistry, Department of Internal Medicine, Eberhard Karls University Tübingen, Tübingen, Germany; 4 Program in Molecular Medicine, University of Massachusetts Medical School, Worcester, Massachusetts, United States of America; 5 Research Unit Molecular Epidemiology, Institute of Epidemiology, Helmholtz Centre Munich, German Research Center for Environmental Health, Neuherberg, Germany; 6 Department of Molecular Epidemiology, German Institute of Human Nutrition Potsdam-Rehbruecke, Nuthetal, Germany; 7 Department of Epidemiology, German Institute of Human Nutrition Potsdam-Rehbruecke, Nuthetal, Germany; 8 Division of Nutritional and Preventive Medicine, Department of Internal Medicine, Eberhard Karls University Tübingen, Tübingen, Germany; Johns Hopkins Bloomberg School of Public Health, United States of America

## Abstract

Mitogen-activated protein kinase kinase kinase kinase 4 (MAP4K4) is expressed in all diabetes-relevant tissues and mediates cytokine-induced insulin resistance. We investigated whether common single nucleotide polymorphisms (SNPs) in the *MAP4K4* locus associate with glucose intolerance, insulin resistance, impaired insulin release, or elevated plasma cytokines. The best hit was tested for association with type 2 diabetes. Subjects (N = 1,769) were recruited from the Tübingen Family (TÜF) study for type 2 diabetes and genotyped for tagging SNPs. In a subgroup, cytokines were measured. Association with type 2 diabetes was tested in a prospective case-cohort study (N = 2,971) derived from the EPIC-Potsdam study. Three SNPs (rs6543087, rs17801985, rs1003376) revealed nominal and two SNPs (rs11674694, rs11678405) significant associations with 2-hour glucose levels. SNPs rs6543087 and rs11674694 were also nominally associated with decreased insulin sensitivity. Another two SNPs (rs2236936, rs2236935) showed associations with reduced insulin release, driven by effects in lean subjects only. Three SNPs (rs11674694, rs13003883, rs2236936) revealed nominal associations with IL-6 levels. SNP rs11674694 was significantly associated with type 2 diabetes. In conclusion, common variation in *MAP4K4* is associated with insulin resistance and β-cell dysfunction, possibly via this gene’s role in inflammatory signalling. This variation’s impact on insulin sensitivity may be more important since its effect on insulin release vanishes with increasing BMI.

## Introduction

Type 2 diabetes mellitus represents a major and increasing health problem in the affluent societies of the modern industrialized world. Two pathomechanisms pave the way to chronic hyperglycaemia and overt type 2 diabetes, i.e., insulin resistance and β-cell failure {for review, see [1]}. The latter is considered a late event [2], to be predominantly determined by genetics [3], [4], and to depend on pre-existing insulin resistance [5]. Insulin resistance is one of the earliest hallmarks of the prediabetic state and results from a complex interplay between obesity-favouring environmental factors, such as unrestricted supply of high-caloric foods and markedly increased sedentary activities during work and leisure, on the one hand, and a permissive genetic background, on the other hand [6]. Impaired insulin action causes decreased glucose disposal (by skeletal muscle and adipose tissue) as well as loss of insulin-dependent suppression of hepatic glucose production and adipose tissue lipolysis. All these metabolic consequences favour the establishment of hyperglycaemia and hyperlipidaemia.

Recently, insulin resistance was shown to be accompanied by Kupffer cell activation in the liver [7], T-lymphocyte and macrophage infiltration into adipose tissue and skeletal muscle [8], [9], and a transition in macrophage polarization from an alternative anti-inflammatory M2 state to a classical pro-inflammatory M1 state [10]. Pro-inflammatory cytokines released by M1 macrophages, such as TNF-α and IL-6, are able to induce cellular insulin resistance at the level of insulin receptor/insulin receptor substrate (IRS) tyrosine dephosphorylation [11] as well as IRS serine phosphorylation via several IRS kinases including c-Jun N-terminal kinase [JNK], inhibitor of κB kinase, and protein kinase C [12]. Thus, local and probably also systemic low-grade inflammation is an integral part of the pathogenesis of insulin resistance and type 2 diabetes.

Mitogen-activated protein kinase kinase kinase kinase 4 (MAP4K4; MIM ID *604666), formerly designated hematopoietic progenitor kinase/germinal centre kinase-like kinase (HGK) or non-catalytic region of tyrosine kinase adaptor protein (NCK)-interacting kinase (NIK), belongs to the Sterile 20 (Ste20) family of serine/threonine kinases, is expressed in several tissues (e.g., heart, brain, skeletal muscle, pancreas, liver) and cell types (adipocytes, myocytes, macrophages), and represents a TNF-α-inducible upstream activator of the JNK pathway [13], [14]. Thus, MAP4K4 is involved in inflammatory signalling and is a potential mediator of cytokine-induced cellular insulin resistance. In support of this hypothesis, MAP4K4 was shown, by knockdown experiments, to block insulin-dependent glucose uptake and to mediate TNF-α-triggered cellular responses, such as inhibition of adipogenesis and glucose transporter 4 expression in 3T3-L1 adipocytes [15] and JNK activation, IRS-1 serine phosphorylation, and cellular insulin resistance in human skeletal muscle cells [16].

Due to MAP4K4’s molecular role in inflammation and insulin resistance, we investigated whether common (minor allele frequency [MAF] >0.05) single nucleotide polymorphisms (SNPs) tagging the human *MAP4K4* locus associate with prediabetic traits, such as glucose intolerance, insulin resistance, impaired insulin release, or elevated plasma TNF-α and IL-6 levels in White European subjects at increased risk for type 2 diabetes recruited from the Tübingen family (TÜF) study for type 2 diabetes. The best hit was further tested for association with type 2 diabetes risk in a prospective case-cohort study derived from the European Prospective Investigation into Cancer and Nutrition (EPIC)-Potsdam study.

## Materials and Methods

### Ethics Statement

Informed written consent to the studies was obtained from all participants and the parents of the minors. The studies adhered to the Declaration of Helsinki. The TÜF study protocol was approved by the Ethical Committee of the Medical Faculty of the University of Tübingen, the EPIC-Potsdam study protocol was approved by the Ethical Committee of the State of Brandenburg.

### Subjects

A study population of 1,769 White Europeans was recruited from the ongoing TÜF study that currently encompasses >2,300 participants at increased risk for type 2 diabetes (non-diabetic individuals with family history of type 2 diabetes or diagnosis of impaired fasting glycaemia) [17]. All participants underwent the standard procedures of the study protocol including assessment of medical history, smoking status and alcohol consumption habits, physical examination, routine blood tests, and an oral glucose tolerance test (OGTT). Selection of the present study population was based on the absence of newly diagnosed diabetes and the availability of complete sets of clinical data. The participants were not taking any medication known to affect glucose tolerance or insulin secretion. In particular, the subjects were not taking metformin, thiazolidinediones, steroids, or acetylsalicylic acid. Two-and-a-half percent of the subjects were on lipid-lowering drugs (statins, fibrates, ezetimibe, or a combination thereof) and 10% on antihypertensive drugs (beta blockers, ACE inhibitors, angiotensin II receptor antagonists, diuretics, or a combination thereof; Table S1). All participants abstained from any medication on the day of examination. From the overall population, a subgroup of 502 individuals was randomly selected for TNF-α and IL-6 measurements (3% on lipid-lowering drugs, 11% on antihypertensive drugs; Table S1). The clinical characteristics of the overall population and the subgroup are given in Table 1.

**Table 1:** Clinical characteristics of the study population.

			Overall population (N = 1,769)								Subgroup TNF-α/IL-6 (N = 502)							
			Median			Minimum			Maximum		Median			Minimum			Maximum	
Age (y)			38			14			91		37			15			69	
BMI (kg/m^2^)			27.6			16.3			86.5		26.9			17.4			55.3	
Habitual physical activity score*			8.13			2.27			11.88		8.13			3.51			10.75	
Fasting glucose (mmol/L)			5.10			3.00			7.00		5.06			3.00			6.94	
Glucose 120 min OGTT (mmol/L)			6.17			2.44			11.06		6.06			2.78			11.06	
HOMA-IR (mmol*mU/L^2^)			1.90			0.27			25.52		1.59			0.32			25.52	
ISI OGTT (*10^15^ L^2^/mol^2^)			12.8			1.1			69.7		14.7			1.4			65.1	
AUC_Ins 0–30_/AUC_Glc 0–30_ (*10^−9^)			34.6			6.0			289.1		31.9			7.5			264.9	
AUC_C-Pep 0–120_/AUC_Glc 0–120_ (*10^−9^)			310			91			841		306			109			816	
TNF-α (ng/L)			–			–			–		1.34			0.06			136.00	
IL-6 (ng/L)			–			–			–		0.64			0.02			25.93	
Leukocyte number (µl^−1^)**			6,270			337			18,030		6,120			337			16,200	
C-reactive protein (mg/dL)**			0.18			0.01			4.79		0.10			0.01			4.39	
Total cholesterol (mg/dL)**			189			63			468		187			67			350	
LDL-cholesterol (mg/dL)**			116			15			279		115			17			223	
HDL-cholesterol (mg/dL)**			52			24			138		52			27			110	
	**Overall population (N = 1,769)**									**Subgroup TNF-α/IL-6 (N = 502)**								
	**Gender**			**Glucose tolerance status**						**Gender**			**Glucose tolerance status**					
	**women**	**men**		**NGT**	**IFG**		**IGT**	**IFG+IGT**		**women**		**men**	**NGT**		**IFG**	**IGT**		**IFG+IGT**
Number	1,159	610		1,248	199		174	148		301		201	373		44	48		37
Proportion (%)	65.5	34.5		70.6	11.2		9.8	8.4		60.0		40.0	74.3		8.8	9.5		7.4

AUC – area under the curve; BMI – body mass index; C-Pep – C-peptide; Glc – glucose; HDL – high-density lipoprotein; HOMA-IR – homeostasis model assessment of insulin resistance; IFG – impaired fasting glycaemia; IGT – impaired glucose tolerance; IL – interleukin; Ins – insulin; ISI – insulin sensitivity index; LDL – low-density lipoprotein; NGT – normal glucose tolerance; OGTT – oral glucose tolerance test; TNF – tumour necrosis factor; *available from 1,331 subjects of the overall population and from 446 subjects of the TNF-α/IL-6 subgroup; **available from 1,687 subjects of the overall population and from 493 subjects of the TNF-α/IL-6 subgroup.

The EPIC-Potsdam study includes 27,548 participants, 16,644 women aged mainly 35–65 years and 10,904 men aged mainly 40–65 years, from the general population of Potsdam, Germany, recruited between 1994–1998 [18]. The baseline examination included anthropometric measurements, blood sampling, and a personal interview on lifestyle habits and medical history. Follow-up questionnaires have been administered every 2 to 3 years to obtain information on current medication and newly developed diseases, including diabetes. Potentially incident cases of diabetes were identified in the full cohort in each follow-up questionnaire until August 31^st^ 2005 via self-reports of diabetes diagnosis, diabetes-relevant medication, or dietary treatment due to diabetes. All potentially incident cases of diabetes were verified by questionnaires mailed to the diagnosing physician asking about the date and type of diagnosis, diagnostic tests, and treatment of diabetes. Only subjects with a physicianś diagnosis of type 2 diabetes (ICD10: E11) and a diagnosis date after the baseline examination were considered as confirmed incident cases of type 2 diabetes and included in the study. Within a mean follow-up time of 7.1 years, 849 subjects were confirmed with incident type 2 diabetes. Within the EPIC-Potsdam study, we designed a prospective case-cohort study involving all incident cases and a random sample of 2,500 subjects from the EPIC-Potsdam study population (sub-cohort). After exclusion of participants with a history of diabetes at baseline (self-reported diagnosis, medication or dietary treatment), with self-reported diabetes during follow-up but without the physicianś confirmation, with missing information on anthropometric measurements at baseline, those without blood samples at baseline, and those without fully obtained follow-up data, 2,224 individuals remained in the sub-cohort, and 747 incident cases identified in the rest of the total cohort (not members of the sub-cohort) remained as “external” cases for analyses {for further information about the case-cohort study, see [19]}.

### Assessment of Habitual Physical Activity

One thousand, three hundred and thirty-one participants completed a standardized self-administered and validated questionnaire to measure physical activity [20]. Points were assigned for physical activity at work (work index), at sport during leisure time (sport index), and for physical activities during leisure time other than sport (leisure time index), The habitual physical activity score was calculated as mean points derived from the three indices.

### OGTT in the TÜF Study

A standardized 75-g OGTT was performed after a ten-hour overnight fast, and venous blood samples were drawn at time-points 0, 30, 60, 90, and 120 min for the determination of plasma glucose, insulin, and C-peptide.

### Laboratory Measurements in the TÜF Study

Plasma glucose was determined using a bedside glucose analyser (glucose oxidase method, Yellow Springs Instruments, Yellow Springs, OH, USA). Plasma insulin and C-peptide were measured by commercial immunoassays for ADVIA Centaur, total-, high-density lipoprotein (HDL)-, and low-density lipoprotein (LDL)-cholesterol and wide-range C-reactive protein were measured using the ADVIA 1800 clinical chemical analyser, and blood cell counts were determined on the ADVIA 2120 haematology analyser (all Siemens Healthcare Diagnostics, Eschborn, Germany). Fasting plasma TNF-α and IL-6 were determined by enzyme-linked immunosorbent assays (R&D Systems, Wiesbaden-Nordenstadt, Germany).

### Selection of Tagging SNPs

**Figure 1:**
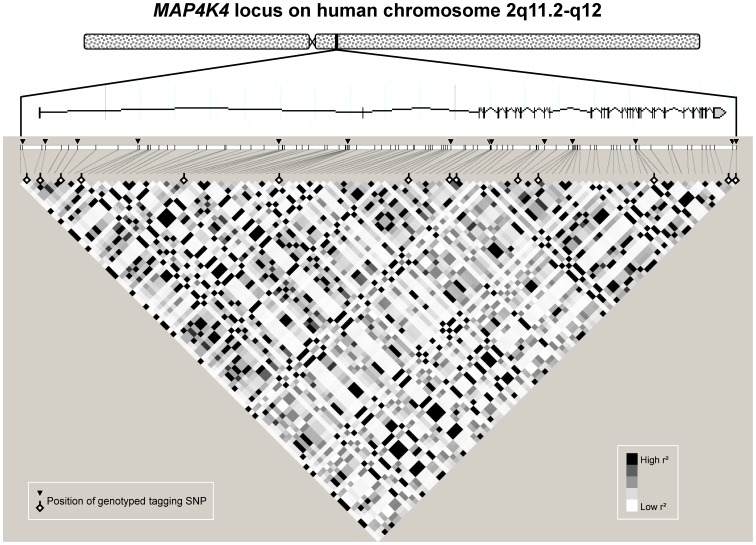
Genomic region of human chromosome 2q11.2-q12 harbouring the *MAP4K4* gene and HapMap linkage disequilibrium data of common (minor allele frequency >0.05) informative single nucleotide polymorphisms (SNPs) within this region. The *MAP4K4* gene consists of 30 exons and 29 introns and spans 196.66 kb from nucleotide position 101,680,920 to nucleotide position 101,877,583. The analysed region additionally included 5 kb of the 5′-flanking region and 3 kb of the 3′-flanking region. This genomic region did not overlap with other known gene loci. The locations of the 14 tagging SNPs (highlighted by flags) are indicated by arrows. Linkage disequilibrium data based on r^2^ values are given by shadings (white diamonds – low linkage; black diamonds – high linkage; grey diamonds – in between).

Based on publicly available phase III data of the International HapMap Project derived from Utah residents with Central European (CEU) ancestry (release #28 August 2010, http://hapmap.ncbi.nlm.nih.gov/index.html.en), we screened *in silico* the complete *MAP4K4* gene spanning 196.66 kb (30 exons, 29 introns) on human chromosome 2q11.2-q12 as well as 5 and 3kb of its 5′- and 3′-flanking regions, respectively (Figure 1). Within this locus, 215 informative HapMap SNPs were present with 106 displaying MAFs >0.05. The HapMap linkage disequilibrium (r^2^) data of the 106 common SNPs are schematically presented in Figure 1. These SNPs reside in non-coding, i.e., intronic or flanking, regions of the gene with one exception: rs1139583 in exon 13 results in the synonymous amino acid exchange Glu418Glu. Among these common HapMap SNPs, 14 SNPs were selected tagging all the other common SNPs within this locus with an r^2^ >0.8 (100% coverage) by Tagger analysis using Haploview software (http://www.broadinstitute.org/scientific-community/science/programs/medical-and-population-genetics/haploview/haploview). The 14 tagging SNPs were rs12465765 (G/A), rs6543087 (A/T), rs11674694 (C/T), rs11894820 (C/T), rs13003883 (T/A), rs17205284 (C/T), rs4851502 (G/A), rs2236936 (C/G), rs2236935 (A/G), rs17801985 (A/G), rs972372 (G/A), rs3771904 (A/T), rs11678405 (T/C), and rs1003376 (G/C).

### Genotyping

In the TÜF study, DNA was isolated from whole blood using a commercial DNA isolation kit (NucleoSpin, Macherey & Nagel, Düren, Germany). Most SNPs were genotyped using Sequenom’s massARRAY System with iPLEX software (Sequenom, Hamburg, German). SNPs rs3771904 and rs1003376 were genotyped by TaqMan assays (Applied Biosystems, Foster City, CA, USA). The TaqMan genotyping reaction was amplified on a GeneAmp PCR system 7000 (50°C for 2 min, 95°C for 10 min, followed by 40 cycles of 95°C for 15 s and 60°C for 1 min), and fluorescence was detected on an ABI Prism sequence detector (Applied Biosystems, Foster City, CA, USA). The Sequenom and TaqMan assays were validated by bidirectional sequencing in 50 randomly selected subjects, and both methods gave 100% identical results. The overall genotyping success rate was 99.6% (rs12465765–99.7%; rs6543087–99.8%; rs11674694–99.6%; rs11894820–99.2%; rs13003883–99.5%; rs17205284–99.7%; rs4851502–99.8%; rs2236936–99.7%; rs2236935–99.7%; rs17801985–99.9%; rs972372–99.2%; rs3771904–99.9%; rs11678405–99.4%; rs1003376–99.7%). Genotyping of the case-cohort study derived from the EPIC-Potsdam study for SNP rs11674694 was performed by KBiosciences (http://www.kbioscience.co.uk) using their own fluorescence-based competitive allele-specific PCR (KASPar).

### Calculations

Homeostasis model assessment of insulin resistance (HOMA-IR) was calculated as {c(glucose[mmol/L])_0_*c(insulin[mU/L])_0_}/22.5 with c = concentration [21]. The insulin sensitivity index derived from the OGTT (ISI OGTT) was estimated as proposed earlier [22]: 10,000/{c(glucose[mmol/L])_0_*c(insulin[pmol/L])_0_*c(glucose[mmol/L])_mean_*c(insulin[pmol/L])_mean_}^½^. OGTT-derived insulin release was estimated by AUC_Ins 0–30_/AUC_Glc 0–30_ and AUC_C-Pep 0–120_/AUC_Glc 0–120_ with AUC = area under the curve, Ins = insulin (in pmol/L), C-Pep = C-peptide (in pmol/L), and Glc = glucose (in mmol/L). AUC_Ins 0–30_/AUC_Glc 0–30_ was calculated as {c(insulin)_0_+c(insulin)_30_}/{c(glucose)_0_+c(glucose)_30_}. AUC_C-Pep 0–120_/AUC_Glc 0–120_ was calculated as ½{½c(C-peptide)_0_+c(C-peptide)_30_+c(C-peptide)_60_+c(C-peptide)_90_+½c(C-peptide)_120_}/½{½c(glucose)_0_+c(glucose)_30_+c(glucose)_60_+c(glucose)_90_+½c(glucose)_120_}. Both indices were recently shown to be superior to several fasting state−/OGTT-derived indices for the detection of genetically determined β-cell failure [23].

### Statistical Analyses

Hardy-Weinberg equilibrium and differences in nominal variables (gender, glucose tolerance status, medication) were tested using χ^2^-test. Linkage disequilibrium (D’, r^2^) was analysed using JLIN, a freeware provided by the Western Australian Institute for Medical Research (http://www.genepi.org.au/jlin). Prior to regression analysis, continuous variables were log*_e_*-transformed in order to approximate normal distribution. Multiple linear regression was performed using the least-squares method. In the regression models, the trait of interest (glucose measures, insulin sensitivity and insulin release indices, plasma cytokines) was chosen as dependent variable, the SNP genotype (in the additive inheritance model) as independent variable, and gender, age, body mass index (BMI), and – whenever testing insulin release – ISI OGTT as confounding variables. Based on testing 14 non-linked tagging SNPs, a p-value <0.0037 was considered statistically significant according to Bonferroni correction for multiple comparisons. We did not correct for the tested traits since they were not independent. Only SNPs with association with prediabetic traits were tested for genotype interactions with age, gender, BMI, and plasma glucose at 120 min of the OGTT by analysis of covariance (ANCOVA). In the ANCOVA (SNP genotypes in the additive inheritance model), two SNPs (rs2236936 and rs2236935) were tested for interaction effects on insulin secretion, five SNPs (rs6543087, rs11674694, rs17801985, rs11678405, rs1003376) for interaction effects on insulin sensitivity, and three SNPs (rs11674694, rs13003883, rs2236936) for interaction effects on plasma IL-6. Thus, Bonferroni-corrected p-values <0.0253, <0.0102, and <0.0170, respectively, were considered statistically significant taking into account the number of SNPs tested in parallel. For the analysis of associations between rs2236936 and rs2236935 and insulin secretion in different BMI strata, a Bonferroni-corrected p-value <0.0253 was chosen as significance threshold. The statistical software package JMP 8.0 (SAS Institute, Cary, NC, USA) was used. Using F-test (one-way ANOVA with fixed effects), the overall study population derived from the TÜF study was sufficiently powered to detect (unadjusted) effect sizes as small as 9.6% (1-β>0.8, α<0.0037, additive inheritance model). The clamp subgroup was sufficiently powered to detect (unadjusted) effect sizes as small as 18.2% (1-β>0.8, α<0.0037, additive inheritance model). Power calculations were performed using G*power 3.0 freeware available at http://www.psycho.uni-duesseldorf.de/aap/projects/gpower/. The relative risk (RR) for SNP rs11674694’s association with risk of type 2 diabetes (in the co-dominant and dominant inheritance models) was calculated as hazard rate ratio with 95% confidence intervals stratified by age using Cox proportional-hazards regression modified according to the Prentice method to account for the case-cohort design. Age was the underlying time variable in the counting processes, with entry defined as the subjects’ age at the time of recruitment and exit defined as age at the diagnosis of diabetes, or censoring. The statistical model was adjusted for the confounding variables gender, waist circumference, and BMI. For this, the statistical software package SAS 9.1 (SAS Institute, Cary, NC, USA) was used, and a two-tailed p-value <0.05 was considered statistically significant.

## Results

### Study Participants

The overall population derived from the TÜF study consisted of 1,769 non-diabetic White European subjects with a median BMI of 27.6 kg/m^2^. Two thirds were women, one third men. About 70% of the subjects were normal glucose tolerant, 30% prediabetic (impaired fasting glycaemia, impaired glucose tolerance, or both at nearly equal proportions). The study participants’ clinical characteristics are given in Table 1.

### Genotyping

All 1,769 participants were genotyped for 14 tagging SNPs of the *MAP4K4* locus (Figure 1) with a total genotyping success rate of 99.6%. All SNPs were in Hardy-Weinberg equilibrium (p≥0.22, all), and the observed MAFs were pretty close to those reported for the HapMap CEU population (Table S2). The genetic linkage between the tagging SNPs ranged from ‘none’ (D’ = 0.00, r^2^ = 0.00) to ‘moderate’ (D’ = 1.00, r^2^ = 0.79, Table 2).

**Table 2:** Linkage disequilibrium data (D’, r^2^) between the 14 tagging SNPs of the *MAP4K4* locus.

SNP	rs12465765	rs6543087	rs11674694	rs11894820	rs13003883	rs17205284	rs4851502	rs2236936	rs2236935	rs17801985	rs972372	rs3771904	rs11678405	rs1003376
rs12465765	–	0.28	0.36	0.01	0.09	0.02	0.01	0.07	0.04	0.05	0.25	0.19	0.03	0.08
rs6543087	1.00	–	0.78	0.09	0.12	0.06	0.05	0.25	0.16	0.18	0.00	0.01	0.36	0.26
rs11674694	1.00	1.00	–	0.02	0.25	0.04	0.04	0.20	0.12	0.15	0.01	0.00	0.46	0.21
rs11894820	1.00	1.00	1.00	–	0.08	0.00	0.00	0.02	0.01	0.01	0.03	0.04	0.01	0.02
rs13003883	0.99	0.62	1.00	0.99	–	0.17	0.15	0.79	0.51	0.19	0.18	0.38	0.12	0.28
rs17205284	1.00	1.00	1.00	1.00	1.00	–	0.01	0.22	0.33	0.03	0.18	0.12	0.02	0.05
rs4851502	1.00	1.00	1.00	0.90	1.00	1.00	–	0.19	0.03	0.03	0.05	0.10	0.02	0.04
rs2236936	1.00	1.00	1.00	1.00	1.00	1.00	1.00	–	0.65	0.15	0.27	0.54	0.09	0.22
rs2236935	0.92	0.97	0.96	1.00	0.98	1.00	0.96	0.99	–	0.11	0.52	0.37	0.06	0.15
rs17801985	1.00	1.00	1.00	0.92	0.98	1.00	0.97	0.99	1.00	–	0.20	0.28	0.07	0.68
rs972372	0.97	0.00	0.11	1.00	0.43	1.00	0.98	0.58	0.99	1.00	–	0.71	0.11	0.29
rs3771904	0.99	0.17	0.02	0.98	0.72	1.00	0.94	0.98	0.99	1.00	1.00	–	0.16	0.40
rs11678405	1.00	0.98	0.99	1.00	0.99	1.00	1.00	0.99	0.98	1.00	0.99	0.99	–	0.10
rs1003376	1.00	0.99	1.00	0.93	0.98	1.00	0.91	0.98	1.00	1.00	1.00	0.99	0.99	–

Values above empty cells represent r^2^ data, values below empty cells D’ data. SNP – single nucleotide polymorphism.

### Associations of SNPs with Plasma Glucose

None of the tested SNPs was associated with fasting plasma glucose levels (Table 3 and Table S3). However, three SNPs (rs6543087, rs17801985, rs1003376) revealed nominal (0.0054≤p≤0.0245) and two SNPs (rs11674694, rs11678405) significant associations (p≤0.0024, both) with plasma glucose levels at 120 min of the OGTT (Table 3). In more detail, the minor alleles of three of these SNPs (rs6543087, rs11674694, rs11678405) were associated with increased, the minor alleles of two SNPs (rs17801985, rs1003376) with decreased 2-hour glucose levels (Table 3).

**Table 3:** Associations between *MAP4K4* SNPs and OGTT-derived metabolic traits (overall population).

	Genotype	N	Fasting glucose(mmol/L)	Glucose 120 minOGTT (mmol/L)	HOMA-IR(mmol*mU/L^2^)	ISI OGTT(*10^15^ L^2^/mol^2^)	AUC_Ins 0–30_/AUC_Glc 0–30_(*10^−9^)	AUC_C-Pep 0–120_/AUC_Glc 0–120_(*10^−9^)
rs6543087	AA	732	5.12±0.57	6.23±1.61	2.80±2.64	15.8±10.9	43.6±33.3	320±107
	AT	813	5.14±0.54	6.42±1.71	2.75±2.47	15.3±10.7	44.7±33.6	324±104
	TT	220	5.13±0.57	6.46±1.58	2.66±2.00	14.2±9.2	44.5±27.4	328±105
p_add_	–	–	0.75	**0.0054**	0.43	**0.0402**	0.35	0.47
rs11674694	CC	858	5.12±0.57	6.22±1.61	2.78±2.60	15.9±11.1	43.9±33.7	322±108
	CT	747	5.15±0.54	6.45±1.70	2.76±2.45	15.0±10.2	44.9±33.1	324±103
	TT	157	5.15±0.56	6.51±1.63	2.66±2.01	14.5±9.9	43.5±26.0	326±107
p_add_	–	–	0.51	0.0024*	0.39	**0.0350**	0.70	0.96
rs2236936	CC	828	5.11±0.55	6.30±1.67	2.72±2.44	15.3±10.5	45.6±34.5	329±109
	CG	741	5.18±0.57	6.47±1.67	2.76±2.44	15.2±10.1	42.1±29.6	315±100
	GG	194	5.04±0.52	6.10±1.48	2.94±2.89	16.7±12.9	46.8±36.5	329±112
p_add_	–	–	0.85	0.90	0.33	0.16	**0.0461**	0.15
rs2236935	AA	1,034	5.11±0.56	6.30±1.69	2.69±2.40	15.4±10.4	44.7±32.9	326±107
	AG	615	5.17±0.56	6.46±1.62	2.86±2.59	15.3±10.6	43.7±32.6	318±104
	GG	114	5.09±0.53	6.21±1.48	2.81±2.73	16.9±12.7	43.6±32.8	322±101
p_add_	–	–	0.24	0.27	0.69	0.30	**0.0419**	0.24
rs17801985	AA	995	5.14±0.55	6.42±1.64	2.80±2.41	15.0±10.6	44.8±30.5	325±104
	AG	661	5.13±0.55	6.27±1.68	2.66±2.47	16.1±10.8	43.3±36.0	320±108
	GG	111	5.14±0.66	6.19±1.66	2.92±3.13	15.2±10.2	44.2±32.2	327±107
p_add_	–	–	0.80	**0.0245**	0.86	0.33	0.98	0.67
rs11678405	TT	1,208	5.12±0.56	6.26±1.64	2.77±2.54	15.6±10.9	44.9±34.3	325±110
	TC	507	5.16±0.54	6.52±1.69	2.74±2.40	15.0±10.2	43.0±29.6	320±97
	CC	44	5.16±0.57	6.73±1.52	2.69±1.92	14.1±9.4	42.3±24.5	314±100
p_add_	–	–	0.10	0.0001*	0.47	0.16	0.31	0.22
rs1003376	GG	799	5.14±0.56	6.43±1.63	2.81±2.48	15.1±10.6	44.4±30.8	323±103
	GC	779	5.12±0.54	6.31±1.66	2.65±2.38	15.9±10.7	43.5±33.9	321±107
	CC	186	5.13±0.61	6.16±1.71	2.90±2.87	15.4±10.4	45.3±33.5	327±111
p_add_	–	–	0.42	**0.0076**	0.85	0.44	0.23	0.46

Data represent means ±SD. Prior to statistical analysis, glucose concentrations and insulin sensitivity measures were adjusted for gender, age, and BMI. Indices of insulin secretion were additionally adjusted for ISI OGTT. p_add_ – p-value additive inheritance model; nominal associations marked by bold fonts; *significant after Bonferroni correction. AUC – area under the curve; BMI – body mass index; C-Pep – C-peptide; Glc – glucose; HOMA-IR – homeostasis model assessment of insulin resistance; Ins – insulin; ISI – insulin sensitivity index; OGTT – oral glucose tolerance test; SNP – single nucleotide polymorphism.

### Associations of SNPs with Insulin Sensitivity

Two of the SNPs associated with increased 2-hour glucose levels (rs6543087, rs11674694) were also nominally associated (0.0350≤p≤0.0402) with decreased insulin sensitivity derived from the OGTT (Table 3). None of the tested SNPs showed association with HOMA-IR (p≥0.30, all, Table 3 and Table S3). To test whether the five SNPs associated with 2-hour glycaemia (Table 3) affect insulin sensitivity depending on genotype interactions with age, gender, BMI, or 2-hour glucose, we performed ANCOVAs. However, none of the SNPs revealed significant interaction effects on insulin sensitivity (p≥0.0154, all).

### Associations of SNPs with Insulin Release

**Figure 2:**
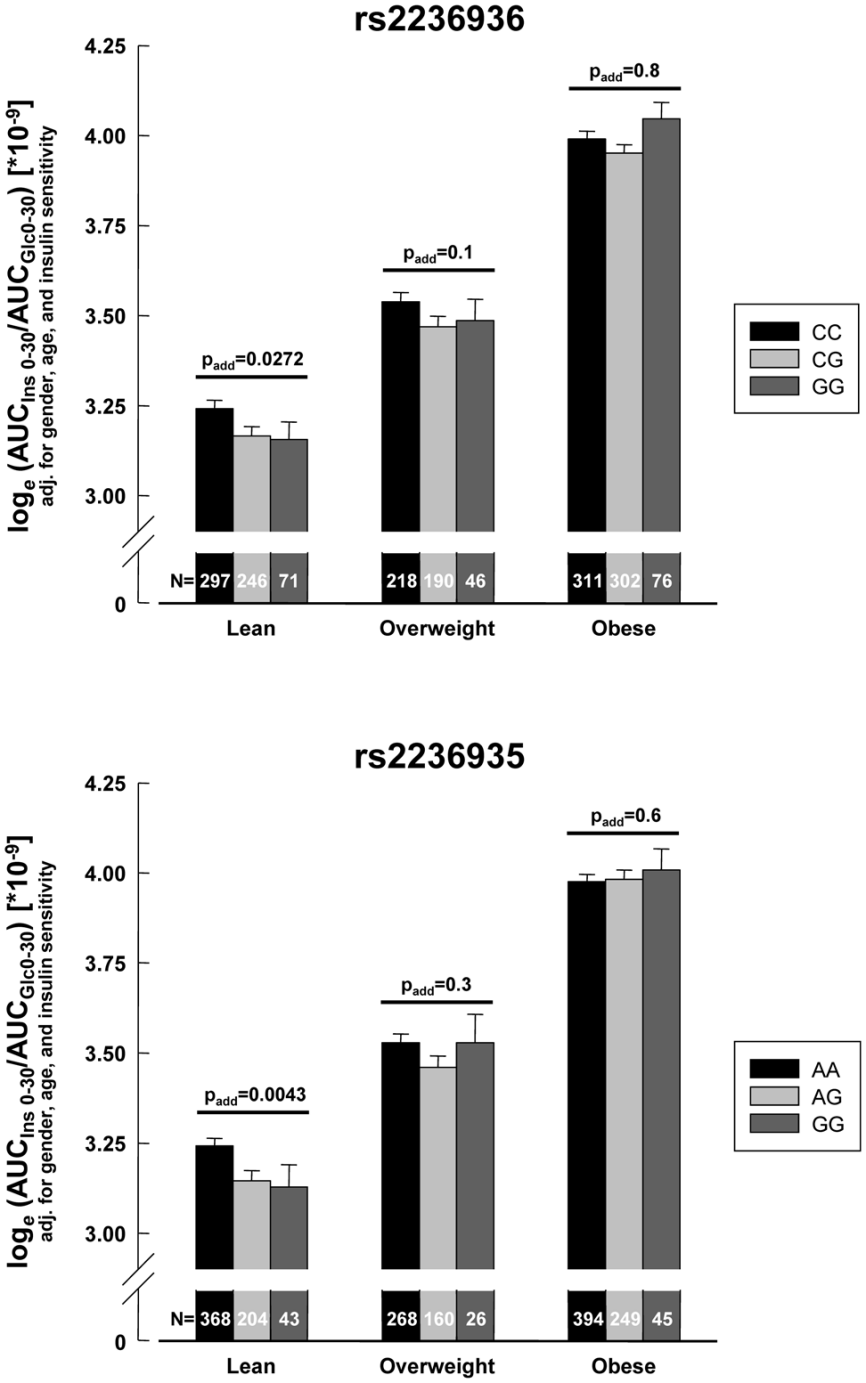
Association of single nucleotide polymorphisms (SNPs) rs2236936 and rs2236935 with insulin release in lean subjects. Subjects were stratified into lean (BMI<25kg/m^2^), overweight (25 kg/m^2^≤BMI<30 kg/m^2^), and obese (BMI≥30 kg/m^2^) subgroups. Insulin release was estimated from the oral glucose tolerance test by calculating the index AUC_Ins 0–30_/AUC_Glc 0–30_ (for calculation, see Materials and Methods). Insulin release was adjusted for gender, age, and insulin sensitivity. Adjusted log*_e_*-transformed data derived from multiple linear regression models ±SE are shown.

Two SNPs not associated with 2-hour glycaemia (rs2236936, rs2236935) showed nominal associations (0.04194≤p≤0.0461) with lower insulin release (AUC_Ins 0–30_/AUC_Glc 0–30_, Table 3). The other SNPs did not reveal associations with insulin release (p≥0.15, all, Table 3 and Table S3). To assess whether the two nominally associated SNPs affect insulin release depending on genotype interactions with age, gender, BMI, or 2-hour glucose, we performed ANCOVAs. SNP rs2236935 revealed significant (p = 0.0096) and SNP rs2236936 nominal (p = 0.0489) interaction with BMI. All other interaction tests were negative (p≥0.08). Therefore, we stratified our overall population in lean (BMI<25 kg/m^2^), overweight (25 kg/m^2^≤BMI<30 kg/m^2^), and obese (BMI≥30 kg/m^2^) subjects and assessed these SNPs’ effects on insulin release in these strata. SNP rs2236935 was significantly (p = 0.0043) and SNP rs2236936 nominally (p = 0.0272) associated with reduced insulin release in lean subjects, whereas no such associations were seen in the other strata (p≥0.1, Figure 2).

### Associations of SNPs with Circulating Cytokines

To address whether *MAP4K4* SNPs affect systemic inflammatory parameters, we measured plasma TNF-α and IL-6 concentrations in 502 randomly selected participants from the overall population. By chance, the subgroup comprised less women (p = 0.0220) and was leaner (p<0.0001, adjusted for gender and age) compared to the overall population, but did not significantly differ in habitual physical activity, fasting glucose levels, 2-hour glucose levels, insulin sensitivity, insulin release, glucose tolerance status, or medication (p≥0.14, all; after appropriate adjustments; subgroup’s clinical characteristics given in Table 1). In this subgroup, three SNPs (rs11674694, rs13003883, rs2236936) revealed nominal associations (0.0094≤p≤0.0252) with plasma IL-6 levels (Table 4). The minor allele of SNP rs11674694 was associated with increased IL-6, whereas the minor alleles of the SNPs rs13003883 and rs2236936 showed association with reduced IL-6 concentrations (Table 4). Taking into account that lipid-lowering and/or antihypertensive medication may have influenced these associations, we additionally adjusted for the drug classes presented in Table S1 by introducing appropriate dummy variables in the regression models. Lipid-lowering, but not antihypertensive, drugs revealed trends towards association with IL-6 in these models (p≤0.08). Independently from drug treatment, SNPs rs11674694 and rs13003883 remained nominally associated with plasma IL-6 levels (p≤0.0239), whereas SNP rs2236936 only retained a trend for association (p = 0.06). The other SNPs were not associated with IL-6 (Table S4). None of the SNPs revealed association with plasma TNF-α (Table 4 and Table S4). The IL-6-associated SNPs did not show significant genotype-age, genotype-gender, genotype-BMI, or genotype-2-hour glucose interactions on plasma IL-6 levels (p≥0.0397, all).

**Table 4:** Associations between *MAP4K4* SNPs and TNF-α/IL-6 (subgroup).

	Genotype	N	TNF-α (ng/L)	IL-6 (ng/L)
rs11674694	CC	238	3.65±11.03	0.91±1.78
	CT	224	2.74±4.68	1.02±1.57
	TT	37	2.47±3.22	1.27±1.89
p_add_	–	–	0.91	**0.0127**
rs13003883	TT	199	3.06±6.24	1.09±1.74
	TA	242	2.48±4.51	0.95±1.80
	AA	61	6.07±18.70	0.84±0.94
p_add_	–	–	0.34	**0.0094**
rs2236936	CC	244	2.81±5.77	1.04±1.61
	CG	212	3.23±10.28	0.99±1.93
	GG	44	4.67±9.26	0.68±0.49
p_add_	–	–	0.08	**0.0252**

Data represent means ±SD. Prior to statistical analysis, cytokine levels were adjusted for gender, age, and BMI. p_add_ – p-value additive inheritance model; nominal associations marked by bold fonts. SNP – single nucleotide polymorphism.

From 1,687 subjects of the overall population, quantitative measurements of total-, LDL-, and HDL-cholesterol, C-reactive protein and leukocyte number were available (Table 1). None of the SNPs associated with 2-hour plasma glucose, insulin sensitivity, insulin secretion, or IL-6 was significantly associated with any of these inflammatory/atherogenic parameters (p≥0.04, all). SNP rs6543087 (which was not associated with IL-6) was the only SNP showing nominal association with one of these traits, i.e., leukocyte number (p = 0.0392).

### Association of SNP rs11674694 with Risk of Type 2 Diabetes in EPIC-Potsdam

The minor T-allele of SNP rs11674694 was nominally associated with increased plasma IL-6 and reduced insulin sensitivity and, more importantly, significantly associated with increased 2-hour blood glucose and, thus, represented the most promising risk allele for the promotion of hyperglycaemia and type 2 diabetes. Therefore, we tested this hypothesis by genotyping the SNP in the EPIC-Potsdam-derived prospective case-cohort study. The genotype distribution was in compliance with the Hardy-Weinberg equilibrium (p>0.05). After adjustment for gender, age, waist circumference, and BMI, heterozygous (RR = 1.248 [0.990–1.573], p = 0.06) and homozygous (RR = 1.254 [0.882–1.782], p = 0.2) T-allele carriers tended to have a higher type 2 diabetes risk compared to homozygous carriers of the major C-allele (co-dominant inheritance model). This trend reached significance in the dominant inheritance model where both T-allele-containing groups were combined (TC+TT) and compared to homozygous C-allele carriers (RR = 1.249 [1.004–1.554], p = 0.0462). This association was only slightly attenuated when additionally adjusted for history of hypertension (diagnosis, medication, or high measured blood pressure) and sport activity level, even though it did not reach statistical significance any longer (RR = 1.228 [0.990–1.523], p = 0.0614).

## Discussion

Here, we report robust associations of five genetically non-linked *MAP4K4* tagging SNPs (rs6543087, rs11674694, rs17801985, rs11678405, rs1003376) with 2-hour plasma glucose levels. The adjusted effect sizes ranged from 0.12 to 0.25 mmol/L (from 2 to 4%) per allele. Upon interrogation of publically available data from the MAGIC consortium (N∼77,000), the effect on 2-hour plasma glucose of one of these SNPs, i.e., rs11678405, was concordant and almost reached nominal significance (p = 0.055, adjusted for BMI only, effect size 0.05 mmol/L per allele). Since 2-hour glucose is commonly used to classify impaired glucose tolerance and type 2 diabetes [24], common genetic variation within *MAP4K4* affects a (pre)diabetes-relevant trait.

In accordance with MAP4K4’s postulated molecular role in inflammation-induced insulin resistance [15], [16], [25], three SNPs (rs11674694, rs13003883, rs2236936) were nominally and specifically associated with altered plasma levels of the inflammatory cytokine IL-6 (adjusted effect sizes 12–20% per allele) and two of the SNPs that increased 2-hour plasma glucose (rs6543087, rs11674694) were also nominally associated with a decrease in insulin sensitivity (adjusted effect sizes ∼4% per allele), as assessed by a well-established dynamic OGTT-derived parameter. Such a reduction in insulin sensitivity SNP carriers face their whole life long could represent the causal mechanism how genetic variation in the *MAP4K4* locus affects 2-hour plasma glycaemia. This, however, needs further replication in larger, comparably phenotyped study populations.

In addition, we could demonstrate that rs11674694, the only SNP showing consistent associations with all three traits, i.e., elevated plasma IL-6, reduced insulin sensitivity, and increased 2-hour glycaemia, was also associated with a 23–25% increase in type 2 diabetes risk (dominant model) in a prospective setting. This raises the question why *MAP4K4* SNPs were not among the top signals for type 2 diabetes detected by array-based genome-wide association (GWA) studies and consortia-driven meta-analyses thereof. This discrepancy might be inherent to differences between hypothesis-driven and hypothesis-free approaches: the hypothesis-free GWA studies provided a series of novel diabetes risk genes with modest effect sizes and largely unknown biological functions, whereas some very strong biological candidate genes with meta-analysis-proven effects on type 2 diabetes risk, such as *CAPN10* and *ENPP1*, have not been replicated by GWA studies. The reasons for the discrepancy regarding *MAP4K4* could be manifold and may result, e.g., from (i) the cross-sectional study design of most GWA studies, (ii) divergent selection criteria for control and/or case groups, (iii) confounders that were not accounted for (ethnicity, environment, prediabetic status), or (iv) the heterogeneity across studies combined for meta-analysis.

Notably, two non-linked *MAP4K4* SNPs (rs2236936, rs2236935) were associated with decreased insulin release (adjusted effect sizes ∼3.5% per allele). This is in keeping with recent *in vitro* observations in rat and human primary β-cells showing that MAP4K4 mediates TNF-α effects, such as reduction of cellular IRS-2 protein, inhibition of proliferation, induction of apoptosis, and inhibition of glucose-stimulated insulin secretion [26], [27]. Interestingly, these SNPs’ associations with impaired β-cell function were driven by their effects in lean subjects only. Why these SNPs’ effects vanish in overweight and obese subjects is currently unknown, but may be explained by obesity-related overriding non-genetic (e.g., environmental) factors.

All SNPs assessed in this study are located in non-coding regions of the *MAP4K4* locus and tag, without exception, non-coding or synonymous common SNPs. Thus, effects of these genetic variants on the function of the MAP4K4 protein are unlikely. Rather, it is conceivable that the SNPs affect *cis*-acting elements, e.g., transcription factor binding sites, thus enhancing or attenuating *MAP4K4* gene transcription. This could also explain the different directions of SNP effects with the minor alleles of three SNPs (rs6543087, rs11674694, rs11678405) increasing and the minor alleles of two SNPs (rs17801985, rs1003376) decreasing 2-hour plasma glycaemia. Different directions of effects were also seen for the associations with plasma IL-6.

Our study has the following limitations: first, due to the limited sample size of the TÜF study, we only analysed common variants with MAFs >0.05; thus, we cannot exclude the existence of rarer variants with direct effects on the MAP4K4’s function (e.g., by amino acid exchange or frameshift mutation); moreover, rarer variants with stronger effects on the gene’s expression and on the tested traits may exist; and secondly, we did not correct our significance level for the number of tested traits because we considered the traits not to be independent; therefore, one or two of our associations may be statistical type I errors; however, the fact that we always found two or more non-linked (i.e., independent) SNPs associated with each trait (2-hour glycaemia, insulin resistance, insulin release, IL-6) clearly argues against spurious findings.

In conclusion, we show here by genetic, but not mechanistic, analysis that common genetic variation in the *MAP4K4* locus is associated with the two major pathomechanisms causing type 2 diabetes, i.e., insulin resistance and β-cell failure, and this is possibly mediated by this gene’s role in inflammatory cytokine signalling. This variation’s impact on insulin sensitivity may be more important with regard to a role in the pathogenesis of type 2 diabetes since its effect on insulin release vanishes with increasing BMI.

## Supporting Information

Table S1 **Lipid-lowering and antihypertensive medication in the study population.**
(DOC)

Table S2 **Minor allele frequencies of the 14** ***MAP4K4*** **tagging SNPs observed in the overall population in comparison to HapMap CEU data.**
(DOC)

Table S3 ***MAP4K4*** **SNPs without associations with OGTT-derived metabolic traits (overall population).**
(DOC)

Table S4 ***MAP4K4*** **SNPs without associations with TNF-α/IL-6 (subgroup).**
(DOC)
