# Supplementary material for: Association of Common Genetic Variants in the MAP4K4 Locus with Prediabetic Traits in Humans
Source: PLoS One. 2012 Oct 18;7(10):e47647. doi: 10.1371/journal.pone.0047647 (PMC3475716; doi:10.1371/journal.pone.0047647)
Supplement: Table S1 — Lipid-lowering and antihypertensive medication in the study population. (DOC) [file pone.0047647.s001.doc]

**Table S1. Lipid-lowering and antihypertensive medication in the study population**

|  | Overall population (N=1,769) | | Subgroup TNF-α/IL-6 (N=502) | |
| --- | --- | --- | --- | --- |
|  | Number | Proportion (%) | Number | Proportion (%) |
| Subjects on lipid-lowering drugs | 45 | 2.5 | 15 | 3.0 |
| - Statins | 39 | 2.2 | 12 | 2.4 |
| - Fibrates | 2 | 0.1 | 0 | 0.0 |
| - Ezetimibe | 3 | 0.2 | 3 | 0.6 |
| - Combination | 1 | <0.1 | 0 | 0.0 |
| Subjects on antihypertensive drugs | 179 | 10.1 | 55 | 11.0 |
| - Beta blockers | 35 | 2.0 | 18 | 3.6 |
| - ACE inhibitors/ATIIR antagonists | 50 | 2.8 | 11 | 2.2 |
| - Diuretics | 10 | 0.6 | 5 | 1.0 |
| - Combination | 84 | 4.7 | 21 | 4.2 |

ACE – angiotensin-converting enzyme; ATIIR – angiotensin II receptor; IL – interleukin; TNF – tumour necrosis factor
