# Supplementary material for: Association of Common Genetic Variants in the MAP4K4 Locus with Prediabetic Traits in Humans
Source: PLoS One. 2012 Oct 18;7(10):e47647. doi: 10.1371/journal.pone.0047647 (PMC3475716; doi:10.1371/journal.pone.0047647)
Supplement: Table S2 — Minor allele frequencies of the 14 MAP4K4 tagging SNPs observed in the overall population in comparison to HapMap CEU data. (DOC) [file pone.0047647.s002.doc]

**Table S2. Minor allele frequencies of the 14 *MAP4K4* tagging SNPs observed in the overall population in comparison to HapMap CEU data**

| SNP | MAF overall cohort | MAF HapMap CEU |
| --- | --- | --- |
| rs12465765 | 0.13 | 0.14 |
| rs6543087 | 0.36 | 0.43 |
| rs11674694 | 0.30 | 0.38 |
| rs11894820 | 0.05 | 0.07 |
| rs13003883 | 0.38 | 0.33 |
| rs17205284 | 0.09 | 0.08 |
| rs4851502 | 0.08 | 0.08 |
| rs2236936 | 0.32 | 0.24 |
| rs2236935 | 0.24 | 0.16 |
| rs17801985 | 0.25 | 0.27 |
| rs972372 | 0.37 | 0.29 |
| rs3771904 | 0.45 | 0.41 |
| rs11678405 | 0.17 | 0.21 |
| rs1003376 | 0.33 | 0.31 |

CEU – descendants from Central Europe; MAF – minor allele frequency; SNP – single nucleotide polymorphism
