# Supplementary material for: Association of Common Genetic Variants in the MAP4K4 Locus with Prediabetic Traits in Humans
Source: PLoS One. 2012 Oct 18;7(10):e47647. doi: 10.1371/journal.pone.0047647 (PMC3475716; doi:10.1371/journal.pone.0047647)
Supplement: Table S3 — MAP4K4 SNPs without associations with OGTT-derived metabolic traits (overall population). (DOC) [file pone.0047647.s003.doc]

**Table S3. *MAP4K4* SNPs without associations with OGTT-derived metabolic traits (overall population)**

|  | Genotype | N | Fasting glucose (mmol/L) | Glucose 120min OGTT (mmol/L) | HOMA-IR (mmol*mU/L^2^) | ISI OGTT  (*10^15^ L^2^/mol^2^) | AUC_Ins 0-30_/ AUC_Glc 0-30_ (*10^-9^) | AUC_C-Pep 0-120_/ AUC_Glc 0-120_ (*10^-9^) |
| --- | --- | --- | --- | --- | --- | --- | --- | --- |
| rs12465765 | GG | 1,327 | 5.14 ±0.56 | 6.34 ±1.65 | 2.76 ±2.53 | 15.7 ±10.8 | 43.5 ±32.4 | 320 ±103 |
|  | GA | 407 | 5.13 ±0.55 | 6.35 ±1.69 | 2.83 ±2.41 | 14.4 ±9.9 | 46.7 ±33.8 | 333 ±111 |
|  | AA | 29 | 5.17 ±0.57 | 6.68 ±1.69 | 2.03 ±1.32 | 17.1 ±11.9 | 41.8 ±33.1 | 312 ±126 |
| p_add_ | - | - | 0.50 | 0.79 | 0.76 | 0.21 | 0.27 | 0.47 |
|  | | | | | | | | |
| rs11894820 | CC | 1,595 | 5.13 ±0.55 | 6.36 ±1.66 | 2.75 ±2.48 | 15.4 ±10.6 | 44.0 ±32.4 | 322 ±105 |
|  | CT | 159 | 5.11 ±0.57 | 6.15 ±1.53 | 2.68 ±2.37 | 15.7 ±10.9 | 45.1 ±34.4 | 329 ±108 |
|  | TT | 1 | 5.11 | 8.61 | 3.10 | 7.8 | 34.6 | 288 |
| p_add_ | - | - | 0.66 | 0.31 | 0.96 | 0.85 | 0.37 | 0.18 |
|  | | | | | | | | |
| rs13003883 | TT | 691 | 5.12 ±0.55 | 6.33 ±1.69 | 2.74 ±2.45 | 15.2 ±10.4 | 45.2 ±34.2 | 327 ±109 |
|  | TA | 819 | 5.16 ±0.56 | 6.41 ±1.66 | 2.75 ±2.46 | 15.3 ±10.3 | 42.9 ±30.8 | 318 ±101 |
|  | AA | 251 | 5.07 ±0.55 | 6.18 ±1.50 | 2.87 ±2.68 | 16.3 ±12.1 | 45.9 ±34.9 | 328 ±109 |
| p_add_ | - | - | 0.92 | 0.71 | 0.37 | 0.16 | 0.22 | 0.61 |
|  | | | | | | | | |
| rs17205284 | CC | 1,455 | 5.13 ±0.56 | 6.34 ±1.67 | 2.72 ±2.43 | 15.5 ±10.7 | 43.8 ±32.7 | 323 ±106 |
|  | CT | 291 | 5.15 ±0.55 | 6.37 ±1.58 | 2.92 ±2.62 | 15.1 ±10.1 | 46.7 ±33.4 | 324 ±106 |
|  | TT | 18 | 5.16 ±0.55 | 6.45 ±1.65 | 3.25 ±4.14 | 16.7 ±14.8 | 38.6 ±27.0 | 279 ±84 |
| p_add_ | - | - | 0.94 | 0.96 | 0.90 | 0.66 | 0.80 | 0.31 |
|  | | | | | | | | |
| rs4851502 | GG | 1,486 | 5.14 ±0.55 | 6.34 ±1.64 | 2.77 ±2.49 | 15.3 ±10.6 | 44.4 ±33.1 | 324 ±106 |
|  | GA | 268 | 5.10 ±0.58 | 6.24 ±1.72 | 2.64 ±2.45 | 16.2 ±11.1 | 42.3 ±30.5 | 314 ±101 |
|  | AA | 12 | 4.94 ±0.39 | 5.68 ±1.33 | 3.74 ±2.96 | 11.0 ±6.9 | 62.2 ±28.2 | 424 ±146 |
| p_add_ | - | - | 0.20 | 0.05 | 0.30 | 0.42 | 0.93 | 0.58 |
|  | | | | | | | | |
| rs972372 | GG | 706 | 5.13 ±0.56 | 6.31 ±1.70 | 2.67 ±2.39 | 15.8 ±10.8 | 43.6 ±32.2 | 322 ±105 |
|  | GA | 806 | 5.14 ±0.55 | 6.35 ±1.62 | 2.81 ±2.56 | 15.0 ±10.0 | 44.3 ±32.8 | 323 ±104 |
|  | AA | 243 | 5.14 ±0.54 | 6.40 ±1.62 | 2.76 ±2.39 | 16.0 ±12.1 | 45.2 ±33.2 | 326 ±110 |
| p_add_ | - | - | 0.96 | 0.56 | 0.76 | 0.92 | 0.65 | 0.99 |
|  | | | | | | | | |
| rs3771904 | AA | 538 | 5.11 ±0.56 | 6.30 ±1.70 | 2.66 ±2.41 | 15.9 ±10.9 | 44.4 ±33.9 | 323 ±105 |
|  | AT | 863 | 5.16 ±0.55 | 6.40 ±1.63 | 2.81 ±2.52 | 15.0 ±9.9 | 43.3 ±31.2 | 322 ±104 |
|  | TT | 367 | 5.10 ±0.55 | 6.28 ±1.64 | 2.78 ±2.52 | 16.0 ±11.9 | 46.2 ±34.4 | 326 ±111 |
| p_add_ | - | - | 0.81 | 0.84 | 0.56 | 0.70 | 0.43 | 0.64 |

Data represent means ±SD. Prior to statistical analysis, glucose concentrations and insulin sensitivity measures were adjusted for gender, age, and BMI. Indices of insulin secretion were additionally adjusted for ISI OGTT. p_add_ – p-value additive inheritance model. AUC – area under the curve; BMI – body mass index; C-Pep – C-peptide; Glc – glucose; HOMA-IR – homeostasis model assessment of insulin resistance; Ins – insulin; ISI – insulin sensitivity index; OGTT – oral glucose tolerance test; SNP – single nucleotide polymorphism
