# Supplementary material for: Association of Common Genetic Variants in the MAP4K4 Locus with Prediabetic Traits in Humans
Source: PLoS One. 2012 Oct 18;7(10):e47647. doi: 10.1371/journal.pone.0047647 (PMC3475716; doi:10.1371/journal.pone.0047647)
Supplement: Table S4 — MAP4K4 SNPs without associations with TNF-α/IL-6 (subgroup). (DOC) [file pone.0047647.s004.doc]

**Table S4. *MAP4K4* SNPs without associations with TNF-α/IL-6 (subgroup)**

|  | Genotype | N | TNF-α (ng/L) | IL-6 (ng/L) |
| --- | --- | --- | --- | --- |
| rs12465765 | GG | 385 | 3.36 ±9.20 | 0.92 ±1.51 |
|  | GA | 109 | 2.52 ±3.85 | 1.19 ±2.25 |
|  | AA | 6 | 1.11 ±0.35 | 1.43 ±0.94 |
| p_add_ | - | - | 0.93 | 0.18 |
|  | | | | |
| rs6543087 | AA | 198 | 3.34 ±7.43 | 0.93 ±1.91 |
|  | AT | 247 | 3.25 ±9.63 | 1.00 ±1.52 |
|  | TT | 57 | 2.04 ±2.74 | 1.15 ±1.64 |
| p_add_ | - | - | 0.27 | 0.10 |
|  | | | | |
| rs11894820 | CC | 451 | 3.01 ±5.99 | 0.99 ±1.74 |
|  | CT | 48 | 4.48 ±19.56 | 0.98 ±1.11 |
|  | TT | 0 | - | - |
| p_add_ | - | - | 0.09 | 0.28 |
|  | | | | |
| rs17205284 | CC | 432 | 3.25 ±8.76 | 1.02 ±1.79 |
|  | CT | 67 | 2.53 ±4.11 | 0.79 ±0.77 |
|  | TT | 1 | 1.40 | 1.64 |
| p_add_ | - | - | 0.98 | 0.13 |
|  | | | | |
| rs4851502 | GG | 418 | 2.87 ±5.93 | 1.04 ±1.82 |
|  | GA | 81 | 4.57 ±15.55 | 0.78 ±0.79 |
|  | AA | 3 | 3.19 ±3.60 | 0.48 ±0.24 |
| p_add_ | - | - | 0.42 | 0.07 |
|  | | | | |
| rs2236935 | AA | 314 | 3.20 ±9.24 | 1.00 ±1.49 |
|  | AG | 161 | 2.72 ±5.36 | 1.03 ±2.15 |
|  | GG | 26 | 5.16 ±10.49 | 0.66 ±0.44 |
| p_add_ | - | - | 0.16 | 0.16 |
|  | | | | |
| rs17801985 | AA | 280 | 3.22 ±9.35 | 0.94 ±1.08 |
|  | AG | 187 | 3.08 ±6.53 | 1.08 ±2.42 |
|  | GG | 35 | 2.94 ±7.37 | 0.98 ±0.77 |
| p_add_ | - | - | 0.76 | 0.83 |
|  | | | | |
| rs972372 | GG | 223 | 3.44 ±10.64 | 0.82 ±0.72 |
|  | GA | 220 | 2.80 ±5.27 | 1.20 ±2.41 |
|  | AA | 56 | 3.39 ±7.34 | 0.82 ±0.63 |
| p_add_ | - | - | 0.21 | 0.86 |
|  | | | | |
| rs3771904 | AA | 168 | 2.86 ±6.31 | 0.87 ±0.73 |
|  | AT | 244 | 3.30 ±9.83 | 1.15 ±2.31 |
|  | TT | 89 | 3.29 ±6.79 | 0.80 ±0.65 |
| p_add_ | - | - | 0.11 | 0.22 |
|  | | | | |
| rs11678405 | TT | 335 | 3.24 ±9.52 | 0.98 ±1.89 |
|  | TC | 150 | 3.07 ±5.09 | 1.08 ±1.26 |
|  | CC | 14 | 2.08 ±2.03 | 0.47 ±0.27 |
| p_add_ | - | - | 0.82 | 0.13 |
|  | | | | |
| rs1003376 | GG | 212 | 3.52 ±10.50 | 0.96 ±1.16 |
|  | GC | 235 | 2.74 ±5.52 | 1.04 ±2.20 |
|  | CC | 53 | 3.51 ±8.49 | 0.92 ±0.72 |
| p_add_ | - | - | 0.41 | 0.52 |

Data represent means ±SD. Prior to statistical analysis, cytokine levels were adjusted for gender, age, and BMI. p_add_ – p-value additive inheritance model. SNP – single nucleotide polymorphism
